# Fabrication of Long-Term Underwater Superoleophobic Al Surfaces and Application on Underwater Lossless Manipulation of Non-Polar Organic Liquids

**DOI:** 10.1038/srep31818

**Published:** 2016-08-23

**Authors:** Jinlong Song, Liu Huang, Yao Lu, Xin Liu, Xu Deng, Xiaolong Yang, Shuai Huang, Jing Sun, Zhuji Jin, Ivan P. Parkin

**Affiliations:** 1 Key Laboratory for Precision and Non-Traditional Machining Technology of Ministry of Education, Dalian University of Technology, Dalian 116024, China; 2 Department of Chemistry, University College London, 20 Gordon Street, London, WC1H 0AJ, UK; 3 Institute of Fundamental and Frontier Sciences, University of Electronic Science and Technology of China, Chengdu 610054, China

## Abstract

Underwater superoleophobic surfaces have different applications in fields from oil/water separation to underwater lossless manipulation. This kind of surfaces can be easily transformed from superhydrophilic surfaces in air, which means the stability of superhydrophilicity in air determines the stability of underwater superoleophobicity. However, superhydrophilic surfaces fabricated by some existing methods easily become hydrophobic or superhydrophobic in air with time. Here, a facile method combined with electrochemical etching and boiling water immersion is developed to fabricate long-term underwater superoleophobic surfaces. The surface morphologies and chemical compositions are investigated. The results show that the electrochemically etched and boiling-water immersed Al surfaces have excellent long-term superhydrophilicity in air for over 1 year and boehmite plays an important role in maintaining long-term stability of wettability. Based on the fabricated underwater superoleophobic surfaces, a special method and device were developed to realize the underwater lossless manipulation of immiscible organic liquid droplets with a large volume. The capture and release of liquid droplets were realized by controlling the resultant force of the applied driving pressure, gravity and buoyancy. The research has potential application in research-fields such as the transfer of valuable reagents, accurate control of miniature chemical reactions, droplet-based reactors, and eliminates contamination of manipulator components.

Lotus leaf has two types of extreme wettability, one is the superhydrophobicity on the upper side, and another is the underwater superoelophobicity on the lower side^1^. Since 2011, several research groups explored the formation mechanism of the underwater superoleophobicity inspired by lotus leaf^2^,^3^,^4^,^5^. They found the superhydrophilic surfaces with micro rough structures in air can be easily transformed into underwater superoleophobic surfaces. When the superhydrophilic surfaces immersed in water, the water wetted the whole surfaces first, then the polar water trapped in the micro structures greatly decreased the contact area between the non-polar oil phase and the solid surfaces, resulting in an underwater superoleophobic property with a high contact angle and low adhesion, which is also an underwater Cassie-Baxter state. As mentioned above, the fabrication of underwater superoleophobic surfaces can be transferred into the fabrication of superhydrophilic surfaces in air. The superhydrophilicity on metal substrate can be easily obtained by constructing micro structures. However, many initial superhydrophilic surfaces with the fabricated micro structures easily become hydrophobic or superhydrophobic in air with time^6^,^7^,^8^,^9^,^10^,^11^. This transition is independent of the fabrication methods. The micro structures obtained by chemical etching^12^, electrochemical etching^13^, chemical oxidation^14^,^15^, laser etching^6^,^9^, and thermal oxidation^8^ all show this transition behavior, resulting in a poor long-term stability of superhydrophilicity. For example, the superhydrophilicity on Al substrates obtained by HCl etching or electrochemical etching can keep no more than 2 days; the superhydrophilicity on Cu substrates obtained by chemical oxidation only keep for 8 days; and the superhydrophilicity on Al substrates obtained by laser etching can keep no more than 8 days. Therefore, a facile method to fabricate superhydrophilic surfaces with long-term stability need to be studied.

The underwater superoleophobic surfaces have a great application prospect in the field of underwater lossless manipulation of non-polar organic liquids. To date, although there are many papers about lossless manipulation of liquids in air by using superhydrophobic surfaces^16^,^17^,^18^,^19^,^20^,^21^,^22^,^23^,^24^,^25^,^26^,^27^, the paper about underwater lossless manipulation of non-polar organic liquids is less. Yong *et al*. realized the *in-situ* transfer oil droplets in water based on underwater superoleophobic surfaces by adding sugar in the water and switching the density of the water solution^28^. This method need to change the characteristic of the environment water, resulting in a complex manipulation process. Ding *et al*. used electrical potential to control the adhesive force of the underwater superoleophobic surfaces and further realize the *in-situ* capture and release of the oil droplets underwater^29^. However, the main capture force in the Ding’s method is the adhesive force between the liquid droplets and the solid surfaces. This kind of adhesive force is very small and no more than 100 μN, resulting in a small operable limiting volume. Since the underwater lossless manipulation of non-polar organic liquids has potential application in research-fields such as the transfer of valuable reagents, accurate control of miniature chemical reactions, droplet-based reactors, and eliminates contamination of manipulator components, a simple route for the *in-situ* lossless controllable manipulation of non-polar organic liquid droplet with a big volume need to be studied.

Here, we first report a new method to fabricate superhydrophilic surfaces on Al substrates with long-term stability for greater than 1 year. We show that the superhydrophilic surfaces are easily transformed into underwater superoleophobic surfaces when immersed in water. Then, using the non-sticky to non-polar organic liquids of the underwater superoleophobic surfaces, a special transfer-pipette device composed of the fabricated underwater superoleophobic surfaces and pressure-generation device is constructed to realize the underwater lossless manipulation of non-polar organic liquids with a large range of volume. The operable limiting volume of the droplet is shown to be determined by the contact angle, interface tension, and density of organic liquid, which even reaches to 1406 μL for peanut oil.

## Results

### Superhydrophilic Al surfaces with long-term stability

**Figure 1:**
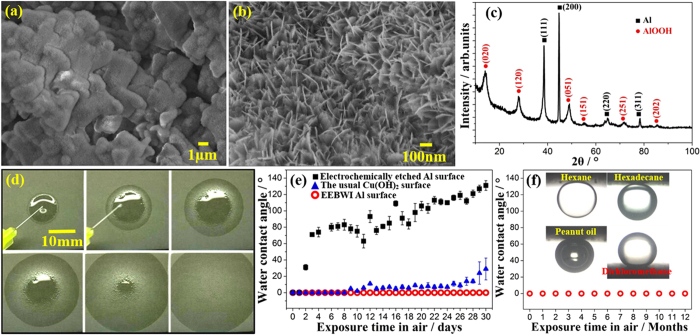
SEM images (**a,b**) and XRD patterns (**c**) of the electrochemically etched and boiling-water immersed (EEBWI) Al surfaces. Spreading processes of water on the EEBWI Al surfaces (**d**). The change of water contact angles on the superhydrophilic surfaces obtained by different processing methods (**e**). The long-time stability of the EEBWI superhydrophilic Al surfaces is more than 1 year (**f**). The insert in (**f**) shows the underwater super-repellent state of non-polar organic liquids on the EEBWI Al surfaces.

Figure 1 shows the micro morphology and chemical composition of the electrochemically etched and boiling-water immersed (EEBWI) Al surfaces. Grain boundaries and dislocations on Al are easily etched by an applied electric field (electrochemical etching) because of their relatively higher energy, forming micrometer-scale rectangular-shaped plateaus and step-like structures with sizes in the range of 1 μm to 5 μm^13^. After immersion in boiling water such surfaces were transformed into nanometer-scale needle-like structures with length of 200 nm and width of 30 nm which covered the whole surfaces, as shown in Fig. 1(a,b). Since the electrochemical etching did not change the surface composition, the diffraction peaks from boehmite (γ-AlOOH, Al_2_O_3_∙H_2_O) were detected beside the diffraction peaks from Al on the XRD patterns after electrochemical etching and immersion in boiling water, thus we learned that the main compositions of the nanometer-scale needle-like structures were boehmite, as shown in Fig. 1(c). The EEBWI Al surfaces showed excellent superhydrophilicity with a very fast spreading velocity when water touched the surfaces, as shown in Fig. 1(d). To analyze the influence of boehmite on the stability of superhydrophilicity, we measured the change of contact angle of the Al surfaces before and after boiling water immersion with the exposure time in air. As shown in Fig. 1(e), the electrochemically etched Al surface without boehmite only kept superhydrophilicity for 1 day, indicating boehmite plays an important role in maintaining superhydrophilicity over a long time period. The EEBWI Al surfaces have considerably better stability than other usual superhydrophilic surfaces, e.g. the usual Cu(OH)_2_ microstructures only kept superhydrophilicity for 8 days^14^,^15^,^30^. The EEBWI Al surfaces even have excellent long-time stability as superhydrophilic surfaces in the air for over 1 year, as shown in Fig. 1(f). When the EEBWI Al surfaces were immersed in water, the polar water became entrapped in between the micro/nanometer-scale structures because of superhydrophilicity, forming a repellent conformal barrier to non-polar organic liquids, resulting in underwater superoleophobicity with small adhesion force (see Supplementary Video S1 and S2). The underwater contact angles of typical organic liquids, e.g. hexane, hexadecane, peanut oil, and dichloromethane, on the EEBWI Al surfaces were larger than 160°. This kind of underwater superoleophobic surface was used in the following transfer-pipette to realize the underwater lossless manipulation of non-polar organic liquids with controllable volume.

### Underwater lossless manipulation of non-polar organic liquids

**Figure 2:**
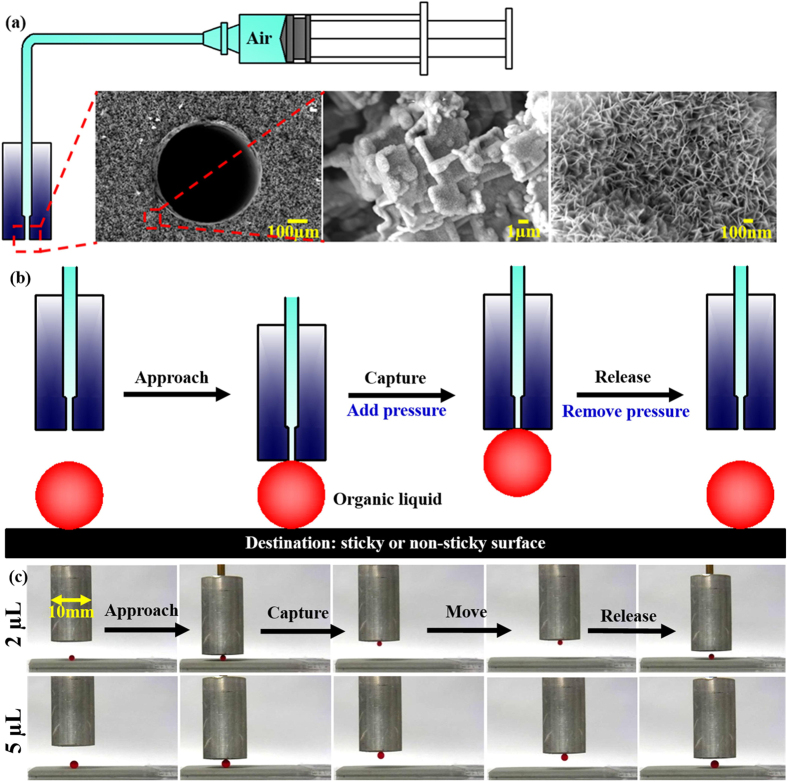
Illustration of the prototype transfer-pipette (**a**) and the complete underwater lossless manipulation processes of non-polar organic liquids (**b**). (**c**) The detailed and practical working processes of underwater lossless manipulation of dichloromethane.

Figure 2(a) shows the illustration of a prototype transfer-pipette, which was mainly composited of underwater superoleophobic hole (EEBWI Al surfaces) and pressure-generation device (e.g. a syringe). The micrometer-scale rectangular-shaped plateaus and step-like structures and nanometer-scale needle-like structures were covered on the end face and inner wall of the hole. The transfer-pipette utilized the air pressure to hold up and capture the organic liquid droplets. No organic liquids residues were left on the transfer-pipette because of the underwater superoleophobic properties of the surface. The captured organic liquids can be released on any surfaces including sticky and non-sticky surfaces. The illustration of the complete underwater lossless manipulation processes of non-polar organic liquid droplets are shown in Fig. 2(b). The transfer-pipette first approached the target organic liquid droplet and then captured the organic liquid droplet under the certain pressure. The captured organic liquid droplet was moved to the destination. Finally, the added pressure was removed and the organic liquid droplet was released. The released organic liquid droplets on non-sticky surfaces can be manipulated again for several times as described in the aforementioned processes. The detailed and practical working processes of underwater lossless manipulation of dichloromethane with volume of 2 μL and 5 μL are shown in Fig. 2(c).

## Discussion

The possible mechanism of the long-term superhydrophilicity was analyzed. Besides N_2_, O_2_, and CO_2_, air also contains a minute amount of organic compounds^9^. Long’s research shows that organic compounds from the surrounding atmosphere will adsorb on the metal surface through the interactions with hydroxyl groups, reducing the content of hydroxyl groups on the metal surface^9^. It is well known that hydroxyl groups are hydrophilic and its content decide the wettability of surface. For electrochemically etched Al surfaces in the air, the decrease of surface hydroxyl groups and adsorption of organic compounds reduce the wettability and increase the contact angle. However, the situation for the EEBWI Al surfaces coated with boehmite is different. Boehmite contains water of crystallization, the content of which is small but far bigger than the hydroxyl groups^31^. The water of crystallization is polar and keeps good affinity for water molecules. In addition, the water of crystallization in the boehmite is very stable in air. All these guarantee the long-term supehrydrophilicity of the EEBWI Al surfaces.

**Figure 3:**
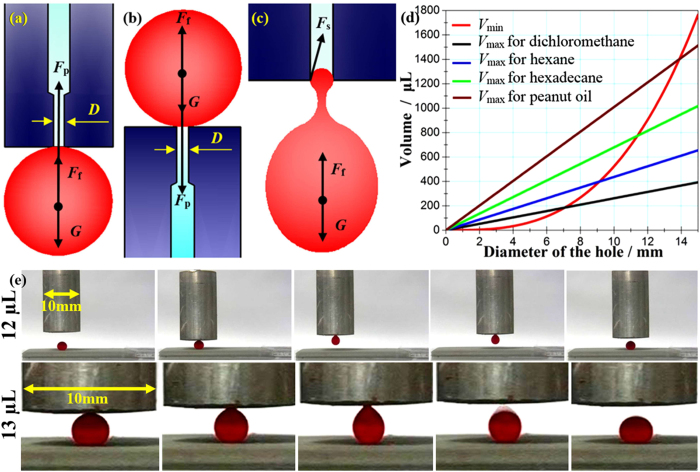
The macro force analysis for a captured droplet (**a**) heavy organic liquids and (**b**) light organic liquids; (**c**) A quasi-stable droplet break from the hole because of the elastic deformation; (**d**) The relationship between the operable volume of the droplet and the size of the hole; (**e**) The transfer-pipette with the hole of 0.5 mm easily manipulated dichloromethane with volume of 12 μL, but could not capture the dichloromethane with volume of 13 μL.

To realize the lossless manipulation, the applied pressure should be smaller than the threshold pressure of organic liquid placed underwater. The threshold pressure is defined as the pressure under which organic liquids pass through the voids in the microstructures on the EEBWI Al surfaces which are filled with water. The threshold pressure *P*_t_ is determined by the corresponding Laplace pressure and can be given by^32^


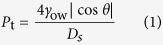


where *γ*_ow_ is the interfacial tensions for organic liquid/water interfaces, *θ* is the underwater contact angle of organic liquid on the EEBEI Al surfaces, and *D*_s_ is the spacing of the voids in the microstructures. Since the spacing of the voids in the microstructures, *D*_s_, is very small (about 5 μm), the threshold pressure *P*_t_ is correspondingly large. Taking dichloromethane as an example of an organic liquid, the values of *γ*_ow_ and *θ* are 28.2 mN/m and 164°, respectively^33^. Then, *P*_t_ = 21.7 kPa. The macro force analysis for a captured droplet is shown in Fig. 3(a,b). For a balance state, the applied force *F*_p_ is determined by


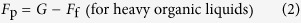



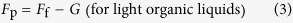


where *G* and *F*_f_ are the gravity and buoyancy of organic liquid, respectively. The densities of heavy and light organic liquids are larger or smaller than the density of water, respectively. The *F*_p_, G, and *F*_f_ are also respectively given by


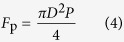



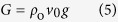



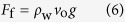


where *P* denotes the applied pressure, *D* the diameter of the hole in the transfer-pipette, *v*_o_ the volume of organic liquid, *ρ*_o_ the density of organic liquid, *ρ*_w_ the density of water, and *g* the acceleration due to gravity.

According to equations (1) to (5), 2, 3, 4, 5, the applied pressure *P* can be calculated as follows,


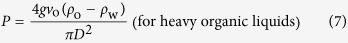



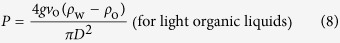


For 5 μL dichloromethane, the values of *ρ*_o_, *ρ*_w_, *V*_o_, *D*, and *g* are 1325 kg/m^3^, 1000 kg/m^3^, 5 μL, 0.5 mm, and 10 N/kg, respectively. Then, *P* = 83 Pa. The applied pressure *P* is far less than the threshold pressure *P*_t_, guarantying the lossless property in the manipulation processes.

The transfer-pipette not only can freely and *in-situ* control the adhesive force to realize the capture and release of liquid droplets, but also can capture the liquid droplets with a large range of volume under the condition of force equilibrium. However, the minimum size of the liquid droplets should be larger than the size of the hole and the value is (πD^3^/6). The maximum size of the liquid droplets is also related with the size of the hole. When the applied force, *F*_p_, captures the top layer of the droplet, if the liquid droplet is very heavy, the droplet will break because of elastic deformation, resulting in a failure manipulation. Theoretically, the volume of a quasi-stable droplet that will eventually break from the hole is mainly determined by competition between the gravity and buoyancy of the pendulous droplet and the vertical component of capillary force, *F*_s_, around the hole edge, as shown in Fig. 3(c). In the quasi-stable state, the relationship between the aforementioned 3 forces is as follows,


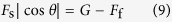



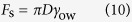


According to equations (5), (6), (9) and (10), the operable maximum volume *V*_max_ of the liquid droplet can be predicted as


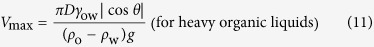



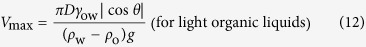


From equations (11) and (12), for a certain liquid, 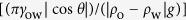 is constant, the operable maximum volume of the droplet is in direct proportion to the diameter *D* of the hole:


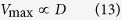


Thus, the operable volume of the liquid droplets is in the range from (πD^3^/6) to 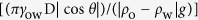. For dichloromethane and the hole with 0.5 mm, according to equation (11), the theoretical operable maximum volume *V*_max_ is about 13.08 μL. Experimentally, the transfer-pipette with a hole of 0.5 mm can easily manipulated the dichloromethane with volume of 12 μL, but could not capture the dichloromethane with volume of 13 μL, as shown in Fig. 3(e) and the Supporting Information (see Supplementary Video S3 and S4), indicating the theoretical results agreed well with the experimental ones.

We also calculated the operable minimum and maximum volume of the droplet for 4 types of liquids, as shown in Table 1. The relationship between the operable volume of the droplet and the size of the hole is shown in Fig. 3(d). Refering to this Figure, we can choose suitable hole size to manipulate the liquid droplet with different types and volume (just like choose a needle for a syringe). Regardless of the hole, for this method, the operable limiting volume of the droplet can be calculated as follows,


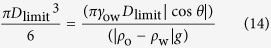



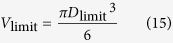


Thus, the operable limiting volume, V_limit_, is 
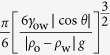
, and the corresponding size of the hole, D_limit_, is 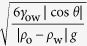. The calculated operable limiting volumes for hexane, hexadecane, peanut oil, and dichloromethane are 187, 776, 395, and 1406 μL, respectively. The corresponding needed limiting sizes of the hole are 7.1, 11.4, 9.1, and 13.9 mm, respectively. Obviously, the operable range of the volume is very large for this method.

**Figure 4:**
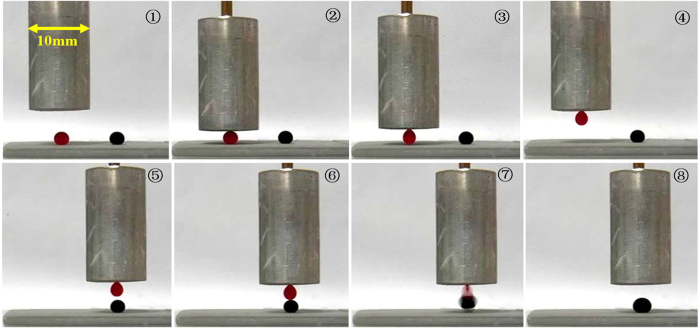
The microdroplet-based micromixing operated by the developed transfer-pipette. Both the red and black droplets are dichloromethane with volume of 10 μL.

The microdroplet-based micromixing was carried out by the developed transfer-pipette, as shown in Fig. 4. The transfer-pipette selectively captured and manipulated one droplet and transferred it to another droplet, completing the mixing of the two microdroplets and demonstrating the potential strategy for quantitative reaction and combinations between microdroplets and components incorporated within microdroplets. This rapid manipulation of the microliter droplets provides us a potential method to save on reactants by ensuring complete liquid transfer and enable microdroplet-based reactions with no loss in sample volumes.

In summary, we developed a new method to fabricate long-term superhydrophilic and underwater superoleophobic surfaces on Al substrates by combining electrochemical etching followed by immersion in boiling water. The new surfaces were covered with the micrometer-scale rectangular-shaped plateaus and step-like structures and nanometer-scale needle-like boehmite structures. The boehmite plays a key role in maintaining long-term superhydrophilicity of the surfaces for over 1 year. Based on the modified Al surfaces, a new type of transfer-pipette mainly composed of an underwater superoleophobic hole and pressure-generation device was developed to realize the underwater lossless manipulation of non-polar organic liquids. The operable minimum volume of the droplet is only determined by the hole size, while the operable maximum volume of the droplet is determined by the hole size, contact angle, interface tension, and density of organic liquid. The relationship between the operable volume of the droplet and the size of the hole can be determined and drawn into a Reference Figure. According to the Reference Figure, it is possible to quickly choose the suitable hole size (just like choosing a needle for a syringe) to manipulate the liquid droplet with different types and volume. The operable limiting volume of the droplet for this method is determined by the contact angle, interface tension, and density of organic liquid, which even reaches to 1406 μL for peanut oil, showing a large operable range in volume. This method can improve the sample volume transfer accuracy, reduce the sample liquid retention, and realize the quantitative microdroplets reaction. This in turn offers the potential for saving expensive reagents, and enabling better quantitative accuracy for transfer of immiscible organic liquids underwater.

## Methods

### Fabrication of underwater superoleophobic surfaces

Two holes with diameter of 0.5 mm and 2 mm and depth of 5 mm and 15 mm were drilled on the two end faces of an aluminum (Al) rod (purity > 99%, 20 mm length, 10 mm diameter), respectively. The end face with 0.5 mm hole was then polished using #1200 and #1500 abrasive paper and electrochemically etched at 500 mA/cm^2^ current density and 6 min processing time in 0.1 mol/L aqueous NaCl solution. Then, the electrochemically etched Al rod was immersed in boiling water for 1 h. The electrochemically etched and boiling water immersed Al surfaces show superhydrophilic in air and superoleophobic under water. To characterize the superhydrophilic and underwater superoleophobic surface better, the plane Al plates with size of 30 × 40 × 2 mm were treated by the aforementioned electrochemical etching and boiling water immersion processes.

### Design of prototype transfer-pipette

A plastic syringe with volume of 1 mL was connected with the Al rod by installing a pressure soft tube into the 2 mm hole to construct a prototype transfer-pipette. The syringe was used as a pressure-generation device to produce negative pressure in the hole of Al rod. In the processes of manipulation of organic liquid droplet, the centerlines of the hole and liquid droplet should be in a straight line as much as possible.

### Characterization

The microstructures and chemical composition of the sample surfaces were observed by a scanning electron microscope (SEM, JSM.6360LV, Japan) and an X-ray diffractometer (Empyrean, Holland). Water and oil droplet contact angle and sliding angle measurements were performed using an in-house goniometer employing ∼5 μL water and oil droplets. The samples were putted in the culture dish in air for preservation. The sliding angle was defined as the angle at which the liquid drop began to slide on the gradually inclined surface. Hexane, hexadecane, peanut oil and dichloromethane were used in the present study as the non-polar organic liquids.

## Additional Information

**How to cite this article**: Song, J. *et al*. Fabrication of Long-Term Underwater Superoleophobic Al Surfaces and Application on Underwater Lossless Manipulation of Non-Polar Organic Liquids. *Sci. Rep*. **6**, 31818; doi: 10.1038/srep31818 (2016).

## Supplementary Material

Supplementary Information

Supplementary Video S1

Supplementary Video S2

Supplementary Video S3

Supplementary Video S4

## Figures and Tables

**Table 1:** Basic parameters of non-polar organic liquids used in the liquid transfer experiments underwater.

Organic liquids	Hexane	Hexadecane	Peanut oil	Dichloromethane
Density at 25 °C [kg/m^3^]	650	770	910	1325
Interfacial tension with water at 20 °C [mN/m]	49.7^34^	51^35^	29.92^36^	28.2^33^
Underwater contact angle on the EEBWI Al surfaces (°)	168 ± 1.2°	167 ± 3.2°	165 ± 5.5°	164 ± 3.9°
Underwater sliding angle on the EEBWI Al surfaces (°)	3.7 ± 0.6°	4.3 ± 0.7°	3.0 ± 0.5°	3.6 ± 0.7°
The operable minimum volume (μL)	0.523*D*^3^	0.523*D*^3^	0.523*D*^3^	0.523*D*^3^
The operable maximum volume (μL)	43.63*D*	67.85*D*	100.88*D*	26.15*D*
The operable limiting volume (μL)	395	776	1406	187
The corresponding limiting size of the hole (mm)	9.1	11.4	13.9	7.1

## References

[b1] Cheng Q. . Janus interface materials: superhydrophobic air/solid interface and superoleophobic water/solid interface inspired by a lotus leaf. Soft Matter 7, 5948–5951 (2011).

[b2] Hejazi V. & Nosonovsky M. Wetting transitions in two-, three-, and four-phase systems. Langmuir 28, 2173–2180 (2011). 22054126 10.1021/la2038284

[b3] Jin M. . Underwater superoleophilicity to superoleophobicity: role of trapped air. Chem. Commun. 48, 11745–11747 (2012). 10.1039/c2cc34805e 23113322

[b4] Liu X. . Clam’s Shell Inspired High‐Energy Inorganic Coatings with Underwater Low Adhesive Superoleophobicity. Adv. Mater. 24, 3401–3405 (2012). 22648962 10.1002/adma.201200797

[b5] Hejazi V., Nyong A. E., Rohatgi P. K. & Nosonovsky M. Wetting transitions in underwater oleophobic surface of brass. Adv. Mater. 24, 5963–5966 (2012). 22945753 10.1002/adma.201202516

[b6] Kietzig A., Hatzikiriakos S. G. & Englezos P. Patterned superhydrophobic metallic surfaces. Langmuir 25, 4821–4827 (2009). 19267439 10.1021/la8037582

[b7] Basu M. . Fabrication and functionalization of CuO for tuning superhydrophobic thin film and cotton wool. J. Phys. Chem. C 115, 20953–20963 (2011).

[b8] Wang G. & Zhang T. Oxygen adsorption induced superhydrophilic-to-superhydrophobic transition on hierarchical nanostructured CuO surface. J. Colloid Interface Sci. 377, 438–441 (2012). 22484170 10.1016/j.jcis.2012.03.035

[b9] Long J., Zhong M., Zhang H. & Fan P. Superhydrophilicity to superhydrophobicity transition of picosecond laser microstructured aluminum in ambient air. J. Colloid Interface Sci. 441, 1–9 (2015). 25481645 10.1016/j.jcis.2014.11.015

[b10] Geng W., Hu A. & Li M. Super-hydrophilicity to super-hydrophobicity transition of a surface with Ni micro–nano cones array. Appl. Surf. Sci. 263, 821–824 (2012).

[b11] Chang F., Cheng S., Hong S., Sheng Y. & Tsao H. Superhydrophilicity to superhydrophobicity transition of CuO nanowire films. Appl. Phys. Lett. 96, 114101 (2010).

[b12] Qin L. . Achieving excellent anti-corrosion and tribological performance by tailoring the surface morphology and chemical composition of aluminum alloys. Rsc Adv 4, 60307–60315 (2014).

[b13] Song J. . Fabrication of superoleophobic surfaces on Al substrates. J. Mater. Chem. A 1, 14783–14789 (2013).

[b14] Zhang F. . Nanowire‐Haired Inorganic Membranes with Superhydrophilicity and Underwater Ultralow Adhesive Superoleophobicity for High‐Efficiency Oil/Water Separation. Adv. Mater. 25, 4192–4198 (2013). 23788392 10.1002/adma.201301480

[b15] Liu N. . Straightforward oxidation of a copper substrate produces an underwater superoleophobic mesh for oil/water separation. ChemPhysChem 14, 3489–3494 (2013). 24106053 10.1002/cphc.201300691

[b16] Li J. . Facile spray-coating process for the fabrication of tunable adhesive superhydrophobic surfaces with heterogeneous chemical compositions used for selective transportation of microdroplets with different volumes. ACS Appl. Mater. Interfaces 6, 8868–8877 (2014). 24807195 10.1021/am5015937

[b17] Hu Z. . Regulating water adhesion on superhydrophobic TiO2 nanotube arrays. Adv. Funct. Mater. 24, 6381–6388 (2014).

[b18] Wu D. . Curvature‐Driven Reversible *In Situ* Switching Between Pinned and Roll‐Down Superhydrophobic States for Water Droplet Transportation. Adv. Mater. 23, 545–549 (2011). 21254261 10.1002/adma.201001688

[b19] Seo J. . Gas‐driven ultrafast reversible switching of super‐hydrophobic adhesion on palladium‐coated silicon nanowires. Adv. Mater. 25, 4139–4144 (2013). 23733597 10.1002/adma.201300979

[b20] Li J., Liu X., Ye Y., Zhou H. & Chen J. Gecko-inspired synthesis of superhydrophobic ZnO surfaces with high water adhesion. Colloids Surf., A 384, 109–114 (2011).

[b21] Hong X., Gao X. & Jiang L. Application of superhydrophobic surface with high adhesive force in no lost transport of superparamagnetic microdroplet. J. Am. Chem. Soc. 129, 1478–1479 (2007). 17243677 10.1021/ja065537c

[b22] Gong G. . Bio-inspired adhesive superhydrophobic polyimide mat with high thermal stability. J. Mater. Chem. 22, 8257–8262 (2012).

[b23] Huang J. . Controllable wettability and adhesion on bioinspired multifunctional TiO 2 nanostructure surfaces for liquid manipulation. J. Mater. Chem. A 2, 18531–18538 (2014).

[b24] Yu X., Zhong Q., Yang H., Wan L. & Xu Z. Mussel-Inspired Modification of Honeycomb Structured Films for Superhydrophobic Surfaces with Tunable Water Adhesion. J. Phys. Chem. C 119, 3667–3673 (2015).

[b25] Liu K., Du J., Wu J. & Jiang L. Superhydrophobic gecko feet with high adhesive forces towards water and their bio-inspired materials. Nanoscale 4, 768–772 (2012). 22139414 10.1039/c1nr11369k

[b26] Cheng Z. . Super-hydrophobic surface with switchable adhesion responsive to both temperature and pH. Soft Matter 8, 9635–9641 (2012).

[b27] Li J. . UV/mask irradiation and heat induced switching on–off water transportation on superhydrophobic carbon nanotube surfaces. Surf. Coat. Technol. 258, 142–145 (2014).

[b28] Yong J. . Reversible Underwater Lossless Oil Droplet Transportation. Adv. Mater. Inter. 2 (2015).

[b29] Ding C. . PANI nanowire film with underwater superoleophobicity and potential-modulated tunable adhesion for no loss oil droplet transport. Soft Matter 8, 9064–9068 (2012).

[b30] Dai C. . Fast formation of superhydrophobic octadecylphosphonic acid (ODPA) coating for self-cleaning and oil/water separation. Soft Matter 10, 8116–8121 (2014). 25177922 10.1039/c4sm01616e

[b31] Majzlan J., Navrotsky A. & Casey W. H. Surface enthalpy of boehmite. Clays Clay Miner. 48, 699–707 (2000).

[b32] Mates J. E., Schutzius T. M., Qin J., Waldroup D. E. & Megaridis C. M. The fluid diode: tunable unidirectional flow through porous substrates. ACS Appl. Mater. Interfaces 6, 12837–12843 (2014). 24988368 10.1021/am5028204

[b33] Pisani E. . Tuning microcapsules surface morphology using blends of homo-and copolymers of PLGA and PLGA-PEG. Soft Matter 5, 3054–3060 (2009).

[b34] Watarai H., Teramae N. & Sawada T. Interfacial Nanochemistry: Molecular Science and Engineering at Liquid-Liquid Interfaces, Springer, 2005.

[b35] Li G., Prasad S. & Dhinojwala A. Dynamic interfacial tension at the Oil/Surfactant-water interface. Langmuir 23, 9929–9932 (2007). 17718527 10.1021/la7014463

[b36] Feuge R. O. Interfacial tension of oil-water systems containing technical mono-and diglycerides. J. Am. Oil Chem. Soc. 24, 49–52 (1947).

