# Supplementary material for: Fabrication of Long-Term Underwater Superoleophobic Al Surfaces and Application on Underwater Lossless Manipulation of Non-Polar Organic Liquids
Source: Sci Rep. 2016 Aug 23;6:31818. doi: 10.1038/srep31818 (PMC4994109; doi:10.1038/srep31818)
Supplement: Supplementary Information [file srep31818-s1.pdf]

Supplementary Information

Fabrication of Long-Term Underwater Superoleophobic Al Surfaces and Application on Underwater Lossless Manipulation of Non-Polar Organic Liquids

Jinlong Song[1,‡], Liu Huang[1,‡], Yao Lu[2], Xin Liu[1,*], Xu Deng[3], Xiaolong Yang[1], Shuai Huang[1], Jing Sun[1], Zhuji Jin[1] & Ivan P. Parkin[2]

[1] Key Laboratory for Precision and Non-Traditional Machining Technology of Ministry of Education, Dalian University of Technology, Dalian 116024, China.

[2] Department of Chemistry, University College London, 20 Gordon Street, London, WC1H 0AJ, UK.

[3] Institute of Fundamental and Frontier Sciences, University of Electronic Science and Technology of China, Chengdu 610054, China.

*Corresponding.xinliu@dlut.edu.cn.

Supplementary Video

Supplementary Video S1: The moving process of dichloromethane on the EEBEI Al surfaces shows very small contact angle hysteresis.

Supplementary Video S2: The touch and departure processes of organic liquid droplets on the EEBEI Al surfaces.

Supplementary Video S3: The dichloromehane with volume of 12 μL can be manipulated.

Supplementary Video S4： The dichloromehane with volume of 13 μL cannot be manipulated.
